# Factors associated with metachronous metastases and survival in locally advanced and recurrent rectal cancer

**DOI:** 10.1002/bjs5.50341

**Published:** 2020-08-28

**Authors:** D. L. H. Baird, C. Kontovounisios, C. Simillis, G. Pellino, S. Rasheed, P. P. Tekkis

**Affiliations:** ^1^ Department of Colorectal Surgery, The Royal Marsden Hospital London UK; ^2^ Department of Surgery and Cancer Imperial College London London UK; ^3^ Department of Colorectal Surgery Chelsea and Westminster Hospital London UK

## Abstract

**Background:**

Better understanding of the impact of metachronous metastases in locally advanced and recurrent rectal cancer may improve decision‐making. The aim of this study was to investigate factors influencing metachronous metastasis and its impact on survival in patients who have a beyond total mesorectal excision (bTME) operation.

**Methods:**

This was a retrospective study of consecutive patients who had bTME surgery for locally advanced and recurrent rectal cancer at a tertiary referral centre between January 2006 and December 2016. The primary outcome was overall survival. Cox proportional hazards regression analyses were performed. The influence of metachronous metastases on survival was investigated.

**Results:**

Of 220 included patients, 171 were treated for locally advanced primary tumours and 49 for recurrent rectal cancer. Some 90·0 per cent had a complete resection with negative margins. Median follow‐up was 26·0 (range 1·5–119·6) months. The 5‐year overall survival rate was 71·1 per cent. Local recurrence and metachronous metastasis rates were 11·8 and 22·2 per cent respectively. Patients with metachronous metastases had a worse overall survival than patients without metastases (median 52·9 months *versus* estimated mean 109·4 months respectively; hazard ratio (HR) 6·73, 95 per cent c.i. 3·23 to 14·00). Advancing pT category (HR 2·01, 1·35 to 2·98), pN category (HR 2·43, 1·65 to 3·59), vascular invasion (HR 2·20, 1·22 to 3·97) and increasing numbers of positive lymph nodes (HR 1·19, 1·07 to 1·16) increased the risk of metachronous metastasis. Nine of 17 patients (53 per cent) with curatively treated synchronous metastases at presentation developed metachronous metastases, compared with 40 of 203 (19·7 per cent) without synchronous metastases (*P* = 0·002). Corresponding median length of disease‐free survival was 17·5 *versus* 90·8 months (*P* < 0·001).

**Conclusion:**

As metachronous metastases impact negatively on survival after bTME surgery, factors associated with metachronous metastases may serve as selection tools when determining suitability for treatment with curative intent.

## Graphical abstract

Surgery for locally advanced and recurrent rectal cancer can achieve good survival outcomes. Some histopathological features significantly increase the number of metachronous metastases and shorten survival. Presentation with a synchronous metastasis that is treated successfully significantly increases the number of metachronous metastases and shortens the time to subsequent metachronous metastasis.

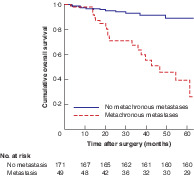

Patient selection is key for bTME

## Abstract (es)

**Antecedentes:**

Una mejor comprensión del impacto de las metástasis metacrónicas en el cáncer de recto localmente avanzado y recidivante puede mejorar la toma de decisiones. El objetivo de este estudio fue investigar los factores que influyen en las metástasis metacrónicas y su impacto en la supervivencia en pacientes sometidos a una operación más amplia que una escisión total del mesorrecto (*beyond total mesorectal excision*, bTME).

**Métodos:**

Se trata de un estudio retrospectivo de pacientes consecutivos sometidos a operaciones bTME por cáncer de recto localmente avanzado y recidivante en un centro de referencia terciario entre enero 2006 y diciembre 2016. El resultado primario fue la supervivencia global. Se realizaron análisis de regresión de riesgos proporcionales de Cox. Se evaluó la influencia de las metástasis metacrónicas en la supervivencia.

**Resultados:**

De un total de 220 pacientes incluidos, 171 fueron tratados por tumores primarios localmente avanzados y 49 por una recidiva de cáncer de recto. Un 90% fue sometido a una resección completa con márgenes negativos. La mediana de seguimiento fue 260 meses (rango 1,5 a 119,6 meses). La supervivencia global a los 5 años fue del 71%. Las tasas de recidiva local y metástasis metacrónicas fueron del 11,8% y del 22,2%, respectivamente. Los pacientes con metástasis metacrónicas presentaron una supervivencia peor en comparación con los pacientes sin metástasis (mediana 46,7 *versus* 109,4 meses, cociente de riesgos instantáneos, *hazard ratio*, HR 6,73, i.c. del 95% 3,23–14,00). Los factores que aumentaron el riesgo de metástasis metacrónicas fueron un estadio T patológico avanzado HR 2,01 (i.c. del 95% 1,35‐2,98), estadio N HR 2,43 (i.c. del 95% 1,65‐3,59), invasión vascular HR 2,20 (i.c. del 95% 1,22‐3,97) y un número creciente de ganglios linfáticos positivos HR 1,19 (i.c. del 95% 1,07‐1,16). En pacientes con metástasis sincrónicas tratadas con intención curativa de inicio, el 52,9% desarrollaron metástasis metacrónicas *versus* el 19,7% en pacientes sin metástasis sincrónicas (*P* = 0,002). La mediana de la supervivencia libre de enfermedad (*disease‐free survival*, DFS) correspondiente fue de 17,5 *versus* 90,8 meses (*P* < 0,0001).

**Conclusión:**

Dado que las metástasis metacrónicas tienen un impacto negativo en la supervivencia tras cirugía bTME, los factores asociados con las metástasis metacrónicas pueden servir como variables de selección para decidir la idoneidad de un tratamiento con intención curativa.

## Introduction

Colorectal cancer is the third most common cancer globally^1^ with significant cancer‐associated mortality^2^. Some 10–20 per cent of patients with rectal cancer present with locally advanced disease^3^, ^4^. In 2013–2014, 9048 patients were diagnosed with rectal cancer in England and Wales, of whom 4978 (55·0 per cent) went on to have a major resection. Of the patients who had a major resection, 251 (5·0 per cent) had known synchronous metastases^5^.

Survival in rectal cancer is improving^6^ owing to advances in imaging^7^, ^8^, surgical technique^9^ and chemoradiotherapy^10^, ^11^. Recurrent cancer heralds a poor prognosis and is most treatable when identified early. Beyond total mesorectal excision (bTME) surgery is used to achieve a complete resection, and is performed in specialist centres. A population‐based study in the USA showed that most patients with locally advanced rectal cancer do not have a bTME resection, despite evidence of improved survival^12^, ^13^.

The bTME consensus statement^4^ defines a bTME operation for locally advanced primary rectal cancer as ‘disease predicted by a magnetic resonance imaging (MRI) scan to require an extended surgical resection beyond the TME plane to achieve a pathological R0 resection’, and in recurrent rectal cancer as ‘the progression or development of new sites of rectal tumour within the pelvis after a previous resection for rectal cancer’^4^. Operations that are bTME are more radical and often require reconstruction with a myocutaneous flap, which increases the complexity of the surgery and commonly necessitates the involvement of an specialist oncoplastic team^13^, ^14^, ^15^. Metastases impact on survival to a greater extent than local recurrence^16^, and a better understanding of metastatic disease may help to personalize choices in treatment^11^, ^17^, ^18^, ^19^, ^20^.

Patients presenting with colorectal metastases were previously not considered for curative resection^21^. Today, in selected patients, survival is comparable to that in patients with non‐metastatic disease^6^, ^22^, ^23^, ^24^. Without active treatment, the median survival is around 5–12 months^25^, ^26^, and 5‐year survival approaches zero^24^, ^25^. Downstaging with combination chemotherapy can render previously inoperable metastatic disease operable^24^, ^27^. Data on metastasis in patients with rectal cancer are restricted, as they are often not recorded on cancer registers^21^.

The aim of this study was to examine the pattern and impact of metachronous metastasis in patients who had bTME surgery for locally advanced primary and recurrent rectal cancer. Metachronous metastases were expected to impact negatively on survival. In addition, prognostic factors for the development of metachronous metastases after bTME rectal cancer surgery were investigated.

## Methods

This was a retrospective study of consecutive patients with locally advanced or recurrent rectal cancer, who underwent a bTME operation with curative intent at the Royal Marsden Hospital, a tertiary referral centre in London, UK, between January 2006 and December 2016. Adult patients were identified from a database. Patients with synchronous metastases that resolved fully on chemoradiotherapy or were completely resected simultaneously with the tumour were included. Patients who presented with metastases that could not be treated with curative intent were excluded. The study was approved by the ethics committee of the institution where it was developed.

Patient evaluation included a history and examination, endoscopy with biopsy, CT of the thorax, abdomen and pelvis (CT‐TAP) and pelvic MRI. If metastases were suspected, PET was performed. Ongoing management plans were agreed on in a specialized bTME multidisciplinary team (MDT) meeting.

Chemoradiotherapy was administered according to European guidelines (irradiation of 45–50·4 Gy in 25–30 fractions over 5 weeks with concomitant chemotherapy of 5‐fluorouracil and capecitabine). Decisions regarding adjuvant chemotherapy were made at the MDT meeting.

Surgery was performed either immediately or 6–8 weeks after neoadjuvant therapy by a consultant‐led team experienced in advanced rectal cancer surgery. Where appropriate, consultant‐led teams in plastic and reconstructive surgery, urology, gynaecology and vascular surgery were involved.

### Definitions, outcomes and data collection

Computerized records for each patient were searched retrospectively. Patient demographics included age, sex, BMI and ASA grade. Pathological characteristics included histopathological tumour (pT) and node (pN) status, vascular invasion (pVI), completeness of resection, total number of lymph nodes in the specimen, number of cancer‐positive lymph nodes, and synchronous metastasis at presentation.

The primary outcome was overall survival. Secondary endpoints included the description of patterns of and risk factors for metachronous metastasis, including the effect of site‐specific metachronous metastases on survival and on single *versus* dual‐site metachronous metastases.

Pelvic exenteration was defined as multivisceral resection of pelvic contents to clear central, anterior, posterior, lateral or inferior compartments as necessary. bTME was defined as an operation for a tumour that extended beyond the circumferential resection margin on preoperative imaging. Overall survival and disease‐free survival were calculated from the date of surgery to the date of death or diagnosis of recurrence respectively. Metastasis was defined as any recurrence outside the bounds of local recurrence. Local recurrence included recurrence at the anastomosis, tumour bed or pelvic lymph nodes. Synchronous metastasis was defined as metastasis presenting at the same time as diagnosis of the primary tumour.

In this institution, each patient with synchronous metastasis was discussed at the colorectal and organ‐specific MDT meeting. An individualized treatment pathway was decided upon for each patient. Surgical treatments included resection of the primary and metastases together, and staged resection of either the primary or metastases first. Metachronous metastasis was defined as metastasis developing after completion of the initial curative treatment. A positive resection margin was defined as the presence of tumour cells at, or within, 1 mm of the resection margin.

### Follow‐up

Routine follow‐up was a surgical outpatient appointment at 2 and 6 weeks, then at 3, 6, 12, 18 and 24 months, and annually thereafter. CT‐TAP and pelvic MRI were done at 6 months and annually thereafter; the serum carcinoembryonic antigen level was measured at each clinic appointment after 6 months; and colonoscopy was performed at 1 and 3 years. Suspected metachronous metastases were discussed by the MDT and at an organ‐specific MDT meeting where required.

### Statistical analysis

All data were collated using Excel® version 15 (Microsoft, Redmond, Washington, USA), and all statistical analyses were performed using SPSS® version 24 (IBM, Armonk, New York, USA).

Differences in demographic factors, postoperative tumour characteristics and treatment modalities between patients with no metachronous metastases and those with metachronous metastases were tested for statistical significance. For categorical variables Pearson's χ^2^ test was used, except where the event rate was less than five, when a two‐sided Fisher's exact test was used. Continuous variables were tested for normality using Shapiro–Wilk test and compared with unpaired *t* tests. Overall and disease‐free survival were analysed with the Kaplan–Meier method, which allowed estimation of outcome in censored data. Comparison between the groups was performed with the log rank Mantel–Cox test for significance, displayed with 95 per cent confidence intervals. Median survival was quoted if it was reached; if not reached, the estimated mean survival was quoted as calculated by the log rank Mantel–Cox test. Hazard ratios (HRs) with 95 per cent c.i. were calculated using Cox proportional hazards regression analysis. Differences between groups were considered significant when the *P* value was 0·050 or less.

**Fig. 1:**
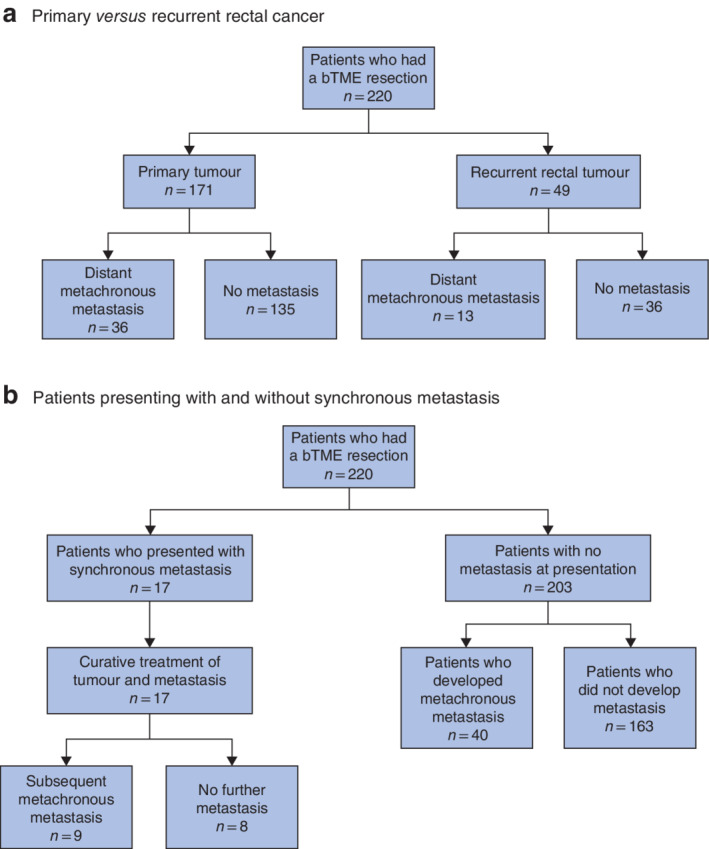
Flow diagrams of the development of metachronous metastasis in patients with locally advanced and recurrent rectal cancer who had a beyond total mesorectal excision operation Development of metachronous metastasis in **a** primary *versus* recurrent rectal cancer and **b** patients presenting with and without synchronous metastasis. bTME, beyond total mesorectal excision.

## Results

A total of 320 patients had undergone a bTME resection, 52 of which included a sacrectomy. Four patients who had a palliative resection, 35 with non‐adenocarcinoma tumours, and 61 who had surgery outside the Royal Marsden Hospital were excluded.

Patient demographics and postoperative tumour characteristics are shown in *Table* *1*. No significant differences were observed between groups in terms of age, sex, BMI or ASA grade. BMI and age were normally distributed according to the Shapiro–Wilk test for normality (both *P* < 0·001).

**Table 1:** Patient demographics and postoperative tumour characteristics

	**Metachronous metastases (*n* = 49)**	**No metachronous metastases (*n* = 171)**	***P*** **‡**
**Age (years)** *	63·7(10·4) (40–80)	61·3(13·1) (27–89)	0·090§
**Sex**			
M	30 (61)	108 (63·2)	0·805
F	19 (39)	63 (36·8)	
**BMI (kg/m** ^**2**^ **)** *	26·6(4·8) (19–43)	26·2(4·4) (18·5–40)	0·721§
**ASA grade**			
I	3 (6)	15 (8·8)	0·740
II	40 (82)	131 (76·6)	
III	6 (12)	25 (14·6)	
**Primary cancer**	36 (73)	135 (78·9)	0·416
**Recurrent cancer**	13 (27)	36 (21·1)	
**pT category**			
pT0	0 (0)	16 (9·4)	0·009
pT1	0 (0)	6 (3·5)	
pT2	2 (4)	28 (16·4)	
pT3	24 (49)	66 (38·6)	
pT4	17 (35)	40 (23·4)	
Unknown	6 (12)	15 (8·8)	
**Lymph node yield** *			
Total	15·4(9·3) (0–26)	16·5(12·0) (1–62)	0·363§
No. of positive nodes	3·3(6·5) (0–26)	0·6(1·4) (0–10)	0·005§
**No metastasis on presentation**	40 (82)	163 (95·3)	0·002
**Synchronous metastasis** **†**	9 (18)	8 (4·7)	

Values in parentheses are percentages unless indicated otherwise;

* values are mean(s.d.) (range).

† Primary tumour and metastasis at first presentation.

‡ Pearson's χ^2^ test, except

§ unpaired *t* test.

**Fig. 2:**
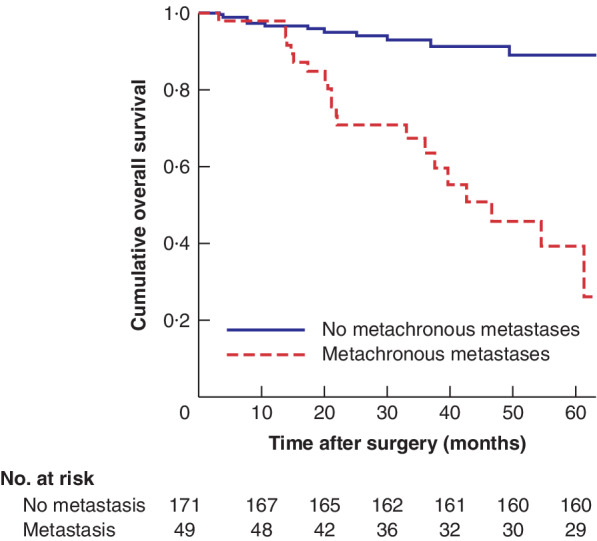
Kaplan–Meier analysis of overall survival in patients with and without metachronous metastasis *P* < 0·001 (log rank Mantel–Cox test).

Metachronous metastasis in patients with primary and recurrent tumours is shown in *Fig*. *1a*. Seventeen patients presented with a bTME tumour and synchronous metastases, and underwent treatment with curative intent (*Fig*. *1b*); they all had neoadjuvant chemoradiotherapy, and 13 also received adjuvant chemotherapy. Nine patients had liver metastases, two patients had ovarian metastases, and one patient each had peritoneal, mesenteric, omental or anal cutaneous metastases. All received a synchronous or *en bloc* resection. Two patients had sacral metastases; one metastasis was resected *en bloc* and the other responded fully to chemoradiotherapy. Nine of these 17 patients (53 per cent) developed subsequent metachronous metastases, compared with 40 of 203 patients (19·7 per cent) without synchronous metastases (*P* = 0·002). Two of these patients died during follow‐up.

### Details of treatment

Treatment details are shown in *Table* *2*. Thirty‐four of 151 patients who underwent pelvic exenteration had an *en bloc* sacrectomy. The ‘bTME other’ group met the criteria for a bTME resection, as 14 had recurrent rectal cancer, 14 had synchronous resections of tumour and metastasis, ten had MRI‐predicted pelvic side wall involvement, 26 had involved and five had threatened circumferential resection margin involvement. There was no 90‐day mortality.

**Table 2:** Details of treatment

	**Metachronous metastases (*n* = 49)**	**No metachronous metastases (*n* = 171)**	***P*** *
Neoadjuvant chemoradiotherapy	41 (84)	138 (80·7)	0·638
Neoadjuvant chemotherapy	1 (2)	5 (2·9)	0·599†
Neoadjuvant radiotherapy	0 (0)	4 (2·3)	0·578†
No chemoradiotherapy	7 (14)	24 (14·0)	0·965
Adjuvant therapy	30 (61)	76 (44·4)	0·117
Exenterative operation	36 (73)	115 (67·3)	0·408
bTME other	13 (27)	56 (32·7)	–

Values in parentheses are percentages. bTME, beyond total mesorectal excision.

* Pearson's χ^2^ test, except

† Fisher's exact test.

**Fig. 3:**
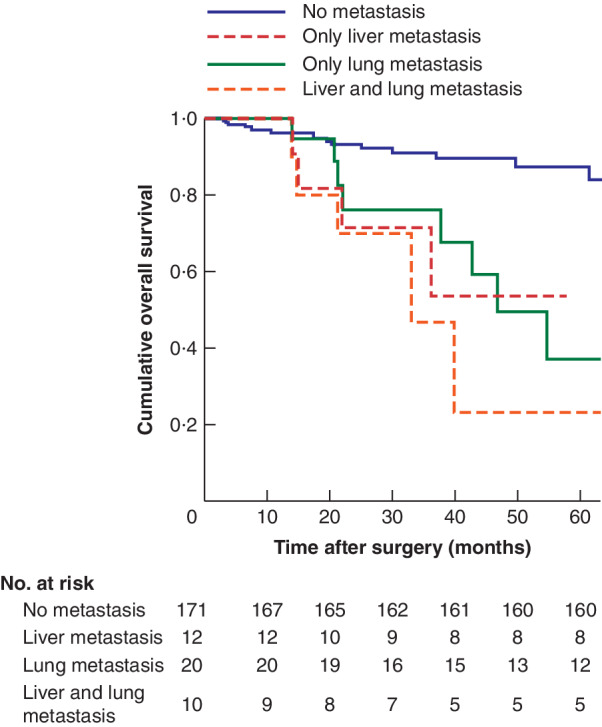
Kaplan–Meier analysis of overall survival in patients with no metachronous metastasis *versus* those with liver, lung, or liver and lung metachronous metastases *P* = 0·616 (log rank Mantel–Cox test).

The overall complete resection rate was 198 of 220 (90·0 per cent). Of the 22 patients who did not have a radical resection, eight (36 per cent) developed metachronous metastases.

### Pattern of metastasis and survival

The median duration of follow‐up was 26·0 (range 1·5–119·6) months. One‐ and 3‐year disease‐free survival rates were 75·4 and 66·7 per cent respectively. Three‐ and 5‐year overall survival rates were 75·0 and 71·1 per cent respectively. In the Cox regression model, pT category (HR 2·01, 95 per cent c.i. 1·35 to 2·98; *P* = 0·001), pN category (HR 2·43, 1·65 to 3·59; *P* < 0·001), pVI (HR 2·20, 1·22 to 3·97; *P* = 0·008) and increasing number of positive lymph nodes (HR 1·19, 1·07 to 1·16; *P* < 0·001) increased the risk of metachronous metastases.

Sixty‐one of the 220 patients (27·7 per cent) had either local recurrence or metachronous metastases at any site. Forty‐nine patients (22·2 per cent) had metachronous metastases (lung, 30; liver, 22; bone, 4; peritoneum, 4; brain, 1; ovary, 1; adrenal, 1). Twenty‐six patients (11·8 per cent) had a local recurrence. Fourteen patients had both local recurrence and distant metastases.

**Fig. 4:**
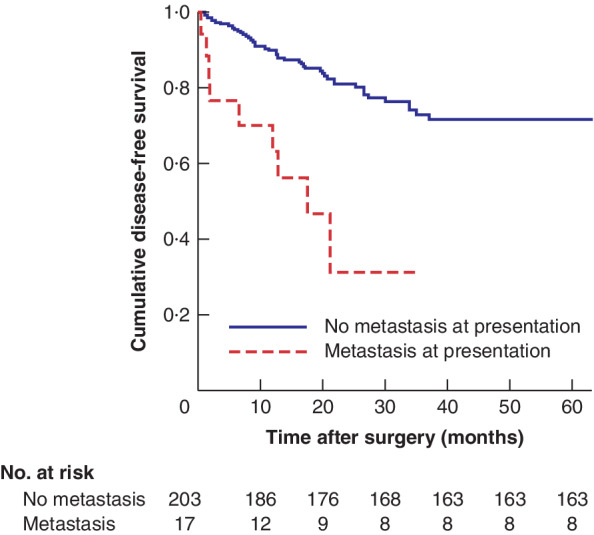
Kaplan–Meier analysis of disease‐free survival in patients who presented with no metastasis *versus* those who presented with synchronous metastases that were treated curatively *P* < 0·001 (log rank Mantel–Cox test).

Metachronous metastases were associated with worse overall survival (*P* < 0·001) (*Fig*. *2*). In the Cox regression model, metachronous metastasis resulted in a worse overall survival (HR 6·73, 95 per cent c.i. 3·23 to 14·00). Mean overall survival was not significantly different in patients with liver, lung, or liver and lung metachronous metastasis (*P* = 0·616) (*Fig*. *3*). In patients presenting with synchronous metastases median overall survival was 52·9 (95 per cent c.i. 42·6 to 63·1) months, compared with an estimated mean overall survival of 109·4 (87·9 to 104·1) months in those without synchronous metastases. Corresponding disease‐free survival was 17·5 (8·6 to 26·4) *versus* 90·8 (83·0 to 98·7) months respectively (*P* < 0·001) (*Fig*. *4*).

## Discussion

Treating bTME rectal cancer is complex, hence information on features that can impact negatively survival is important in assessing suitability for resection, determining the timing of radiotherapy and chemotherapy, and planning postoperative follow‐up. In this series, factors associated with developing metachronous metastases included initial presentation with synchronous metastases, histopathological advancing tumour (pT category) and nodal (pN category) status, the presence of pVI, and an increased number of involved lymph nodes. This study has shown that the presence of metachronous metastases after bTME surgery heralds a significant decrease in survival, as would be expected.

Some 20–30 per cent of patients with synchronous colorectal cancer and liver metastases at presentation can technically be resected^28^; however, actual resection rates are lower at 5–15 per cent^5^, ^29^, ^30^. A population study^26^ of patients with colorectal cancer presenting with metastases noted an overall median survival of 12 months for all patients, 15·3 months in patients who received systemic chemotherapy, and 46·2 months in patients who had metastasectomy. In the present study, patients presenting with a bTME tumour and synchronous metastases undergoing treatment with curative intent developed subsequent metachronous metastases more often and had shorter disease‐free survival than patients presenting with a bTME tumour without metastases. Logically, this should lead to reduced overall survival, but this was not shown. It is important to note that there were only two deaths in the group of 17 patients who presented with synchronous metastases. The small sample size makes the introduction of error likely, and overall survival outcome analysis in this subgroup may not be reliable. The bTME consensus statement^4^ commented on the issue of treating synchronous metastases, noting that it is controversial, the data are sparse, and careful consideration should be given to the suitability of these patients for surgical resection. The statement suggested assessing both the response of the metastases to chemotherapy and the patient's performance status when deciding whether surgery is indicated^4^.

Advanced pT and pN category, the presence of pVI, and the number of involved lymph nodes were all associated with metachronous metastases. These factors have all been shown previously^31^, ^32^, ^33^, ^34^, ^35^ to impact negatively on survival. In pelvic exenteration, previous studies have shown that a positive (R1 or R2) resection margin significantly worsens survival^36^ and significantly increases the local recurrence rate^37^, but has no significant impact on metachronous metastasis^13^. The present study showed that achieving a complete resection did not affect the rate of metachronous metastasis.

This study found no difference in overall survival according to the site of metachronous metastases. The specifics of which single, dual or multiple sites of metachronous metastasis can be treated curatively in combination with a bTME resection are debated, and more work is needed.

Neoadjuvant chemoradiotherapy has been shown to reduce significantly the local recurrence, but not the metachronous metastasis, rate^38^, ^39^. The present study did not show a difference in metastasis rate between patients who received neoadjuvant therapy and those who did not. The extent of surgery (exenterative surgery *versus* bTME other) did not significantly affect the subsequent rate of metachronous metastasis, as has been demonstrated in other studies^13^, ^39^.

In this study, no difference was observed in the rate of metachronous metastasis between selected patients with locally advanced primary rectal cancer and recurrent rectal cancer. This is concordant with the series of Gannon and colleagues^39^, who reported metachronous metastasis rates of 22 and 15 per cent in patients with locally advanced primary rectal cancer and recurrent rectal cancer respectively.

Limitations of the present study include the retrospective design and tertiary referral setting. Multiple factors influence who is referred to a tertiary service, and patients who had a bTME operation were considered carefully, introducing selection bias. Patient numbers were relatively small, so locally advanced and recurrent cancers were analysed together, as is common for studies of locally advanced and recurrent rectal cancer.
